# Prenatal ultrasound diagnosis, intrauterine monitoring and postnatal management of a giant fetal abdominopelvic lymphangioma: a case report and scoping review

**DOI:** 10.3389/fped.2026.1805856

**Published:** 2026-05-07

**Authors:** Matteo Giudice, Milena Viggiano, Chiara Vassallo, Alice Novak, Laura Valfrè, Irma Capolupo, Andrea Dotta, Andrea Conforti, Marco Bonito, Maurizio Guida, Leonardo Caforio, Isabella Fabietti

**Affiliations:** 1 Department of Neurosciences, Reproductive Sciences and Odontostomatology, School of Medicine, Federico II, Naples, Italy; 2 Obstetrics, Gynecology and Maternal Fetal Medicine Unit, Clinical Area of Fetal, Neonatal and Cardiological Sciences, Bambino Gesù Children's Hospital IRCCS, Rome, Italy; 3 Fetal and Neonatal Surgery Unit, Clinical Area of Fetal, Neonatal and Cardiological Sciences, Bambino Gesù Children Hospital IRCCS, Rome, Italy; 4 Neonatal Intensive Care Unit, Bambino Gesù Children's Hospital IRCCS, Rome, Italy; 5 Department of Maternal and Child Health, San Pietro Hospital, Rome, Italy

**Keywords:** fetal abdominal lymphangioma, fetal abdominopelvic lymphangioma, neonatal outcome, pregnancy outcome, prenatal ultrasound diagnosis, pediatric surgery

## Abstract

**Background:**

Fetal lymphangioma is a rare congenital malformation, generally isolated and clinically asymptomatic, except when it invades adjacent tissues, causing compression-related symptoms.

**Objectives:**

To present a rare case of a giant fetal abdominopelvic lymphangioma and to perform, to our knowledge, the first scoping review of the literature specifically addressing prenatal diagnosis, intrauterine monitoring and postnatal management of this condition.

**Methods:**

The PubMed, Scopus and Embase databases were searched up to September 2025. No limitations on the country were made.

**Results:**

We present the case of a 38-year-old primigravida with gestational diabetes, hypothyroidism and severe obesity referred to our institute for a second opinion after prenatal detection of a large multiloculated cystic abdominal mass in the fetus, extending to the pelvis, without vascularization. Follow-up imaging, including a fetal magnetic resonance imaging (MRI), confirmed the suspicion of a giant lymphatic malformation with progressive fetal ascites and associated symptomatic polyhydramnios requiring amnioreduction. A male infant was delivered via emergency cesarean section at 34 + 3 weeks and admitted to neonatal intensive care unit (NICU) with respiratory distress. Due to progressive clinical worsening and persistent fluid accumulation, an abdominal drain was placed, followed by debulking surgery at four months, which confirmed the diagnosis of lymphangioma. Gradual clinical improvement enabled the patient's transfer to the Nutritional Rehabilitation Unit and subsequent discharge. Regarding the scoping review, 20 case reports fulfilled the inclusion criteria. Patients were analyzed concerning the prenatal ultrasound diagnosis of a fetal abdominal lymphangioma, pregnancy outcomes, intrauterine and postnatal treatment of the mass.

**Conclusion:**

Clinical presentation and outcomes of fetal abdominal and abdominopelvic lymphangiomas are highly heterogeneous and strongly influenced by lesion extent and intra-abdominal involvement. Our case, characterized by diffuse intestinal and mesenteric disease, highlights the limitations of curative surgical strategies and underscores the need for early recognition of patients who may require prolonged supportive management rather than a complete excision. The scoping review suggests that while focal lesions are often amenable to surgery with favorable outcomes, diffuse forms often require a multidisciplinary, staged approach, with realistic prognostic counseling and long-term follow-up.

## 1 Introduction

Fetal lymphangioma is a rare congenital malformation that may arise as early as the sixth week of gestation, due either to a failure in the connection between the lymphatic and venous systems or to an abnormal development of the lymphatic channels. These lesions can develop in any region of the body where lymphatic vessels are present. The most common area is the neck (75%), followed by the axillary region (20%), retroperitoneum and abdominal viscera (2%), limbs, bones and mesentery (2%) and cervicomediastinum (1%) (1). Generally, fetal lymphangiomas are isolated findings with a low risk of chromosomal abnormalities or associated malformations and, therefore, are typically associated with favorable clinical outcomes (2).

These malformations may present as unilocular or multilocular cystic structures and can range in diameter from a few millimeters to more than 10 cm. Because of their variable imaging features, they may occasionally be mistaken for dilated bowel loops, localized ascites or anomalies of the urinary tract (3). Although they are typically asymptomatic due to their soft texture, they can invade nearby tissues and cause compression-related symptoms (4).

They are classified into three different types: simple, cavernous and cystic. Simple lymphangiomas consist of small capillary-like channels, typically found in the subcutaneous tissue. Cavernous lymphangiomas consist of larger lymphatic vessels surrounded by fibrous tissue. Cystic lymphangiomas, such as cystic hygromas, are composed of multiple cysts of varying sizes lined with endothelial cells and usually filled with serous or chylous fluid. In some cases, these cysts can become complicated, containing either blood or pus. Mixed lesions, which exhibit characteristics of more than one type, may also occur (5).

The treatment of these lesions is primarily surgical, although sclerotherapy may be used as an initial or adjunctive approach before surgery. Sirolimus may be considered when surgery is not feasible or after failed surgical excision. The feasibility and safety of this treatment were previously reported by Amodeo et al. (2017) in four neonates with giant cystic lymphangiomas, showing only minimal adverse effects (6). The prognosis is generally good, although these tumors can recur, and long-term follow-up is necessary after the primary intervention (7). The available literature primarily consists of isolated case reports that often emphasize technical feasibility rather than providing decision-making frameworks or insights into long-term clinical trajectories. Against this background, the aim of the present case report is not only to describe a complex, refractory case of fetal abdominopelvic lymphangioma, but also to place it in the context of the existing literature. Through what is, to our knowledge, the first scoping review specifically focused on fetal abdominal and abdominopelvic lymphangiomas, we sought to identify key concepts and knowledge gaps, as well as clinically meaningful patterns that may support risk stratification, multidisciplinary management and realistic prognostic counseling for these rare anomalies.

## 2 Case report

A 38-year-old primigravida patient, affected by gestational diabetes, hypothyroidism and severe obesity, was referred to our center of Medicine and Fetal Surgery at Bambino Gesù Pediatric Hospital (OPBG) for a second opinion. The referral followed the detection in the fetus, during a second-trimester screening ultrasound, of an abdominal cystic mass. The lesion measured 57 × 63 mm, appeared multiloculated with internal septations, showed no vascularization on color Doppler imaging and extended caudally into the fetal pelvis. At this stage, the main differential diagnoses included pelvic hemangioma, presacral type IV sacrococcygeal teratoma, pelvic lymphangioma and fetal ovarian cyst.

**Figure 1:**
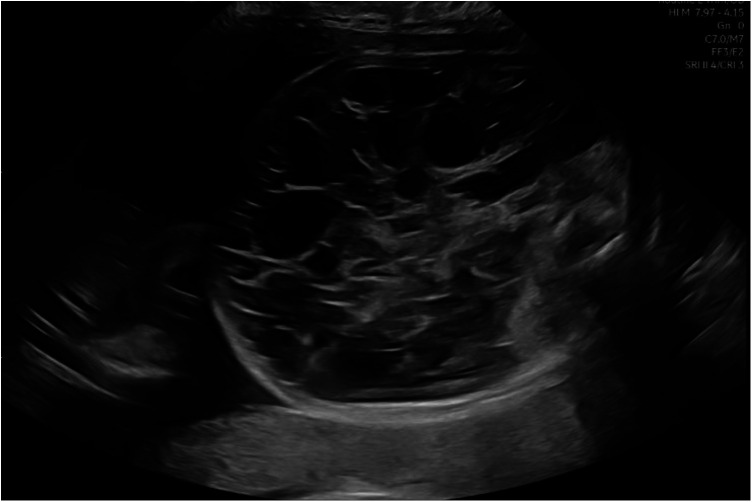
Ultrasound appearance of the lesion at 30 weeks of gestation on an axial scan of the fetal abdomen (Bambino Gesù Children's Hospital, Rome, Italy).

A follow-up ultrasound at 30 weeks of gestation confirmed the presence of a multiloculated cyst, extending from the left hypochondrium to the fetal pelvis with increased dimensions of 99 × 50 × 76 mm (Figure 1).

The remaining fetal anatomy appeared unremarkable, apart from a suspected dilation of intestinal loops, with a maximum diameter of 15 mm.

**Figure 2:**
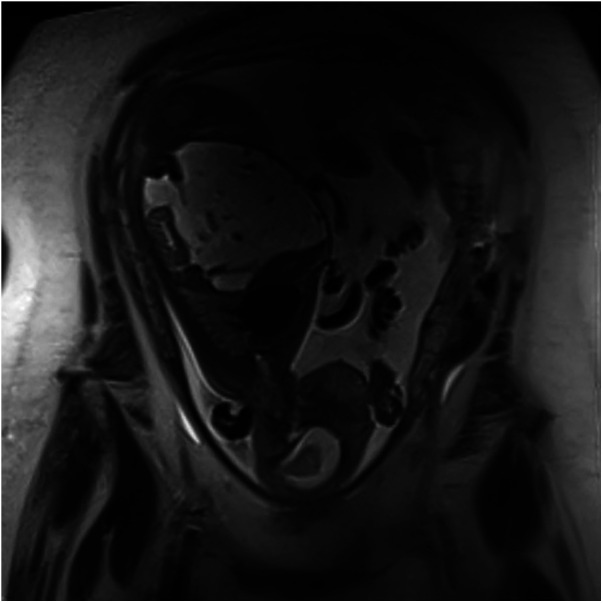
Coronal MRI scan, T2-weighted sequence, showing a hyperintense multiloculated cystic lesion (Bambino Gesù Children's Hospital, Rome, Italy).

**Figure 3:**
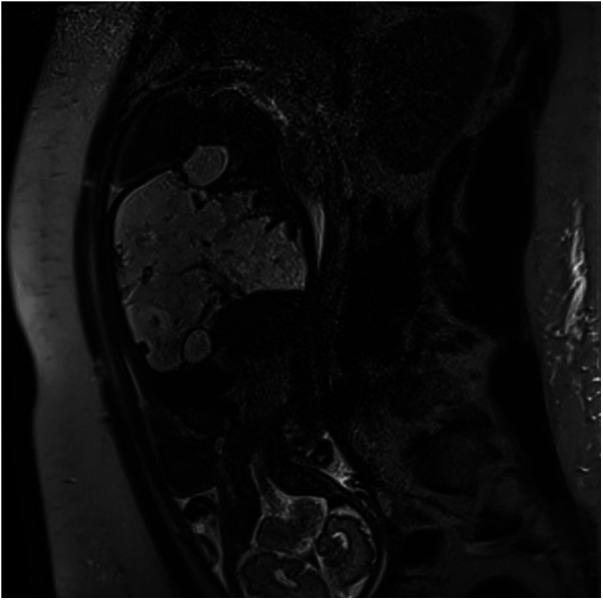
Sagittal MRI scan, T2-weighted sequence, demonstrating the extension of the lesion from the left diaphragmatic dome and the right hepatic margin to the pelvic cavity and transversely across the abdominal cavity (Bambino Gesù Children's Hospital, Rome, Italy).

A fetal magnetic resonance imaging (MRI) was performed to better characterize the ultrasound findings and confirmed the presence of a large multilocular cystic mass occupying all abdominal quadrants, with overall dimensions of approximately 100 × 90 × 80 mm (Figures 2, 3).

Subsequent ultrasound examinations showed a stable lesion size; however, progressive amniotic fluid accumulation was observed, with a maximum vertical pocket of 90 mm, necessitating amnioreduction. Thereafter, the development of progressive perihepatic ascites, increasing from 12.6 mm to 18 mm within one week, prompted the administration of antenatal corticosteroids for fetal lung maturation.

Due to worsening fetal condition, an emergency cesarean section was performed at 34 weeks and 3 days of gestation, resulting in the birth of a male infant weighing 3300 g, with an Apgar score of 6 and 8 at one and five minutes of life, respectively.

Approximately 15 min after birth, the newborn was admitted to the Neonatal Intensive Care Unit (NICU) of OPBG, where he was intubated, sedated and a central venous catheter was placed via a right latero-cervical approach. He required invasive mechanical ventilation for two weeks, followed by non-invasive respiratory support and complete weaning seven days later.

On the second day of life, a percutaneous abdominal drain was placed due to significant daily fluid output, resulting in significant fluid and electrolyte losses that required supplementation.

Postnatal laboratory evaluation demonstrated a consumptive coagulopathy consistent with localized intravascular coagulation, characterized by reduced fibrinogen (141 mg/dL; reference range 187–501 mg/dL), elevated D-dimer (5.96 mg/L FEU; reference range 0.28–1.87 mg/L FEU) and prolonged coagulation parameters (INR 1.6; aPTT 52.5 s; reference range 28.8–47.7 s).

Additional hematological findings included neutrophilia (75.7%; reference range 54%–70%) and marked lymphopenia (6.6%; reference range 22%–34%), the latter persisting throughout the clinical course and likely reflecting lymphatic sequestration within the lymphangioma and associated ascitic fluid.

Biochemical abnormalities were also observed, including hyponatremia (sodium 130 mmol/L; reference range 135–145 mmol/L) and mild hyperkalemia (potassium 5.3 mmol/L; reference range 3.5–5.0 mmol/L), likely secondary to fluid losses.

Finally, elevated cardiac biomarkers (NT-proBNP and high-sensitivity Troponin T) and an increased hemolysis index (478, arbitrary units) were detected, possibly reflecting systemic stress and the hemodynamic impact of the lesion.

Postnatal abdominal MRI confirmed the extension and distribution of the lesion; however, both interventional radiology and neonatal surgery teams deemed the patient unsuitable for sclerotherapy or surgical resection. Given the critical condition and persistent fluid losses, treatment with sirolimus was initiated after multidisciplinary discussion with oncology and vascular anomalies specialists. Serial ultrasounds and MRI demonstrated stable cystic lesions and a marked reduction in ascites, allowing removal of the abdominal drain.

Nevertheless, due to the lack of significant clinical improvement, sirolimus therapy was discontinued following further multidisciplinary evaluation. Given the complexity of the case, an extensive genetic workup was performed, including next-generation sequencing panels for lymphatic anomalies and RASopathies, as well as conventional karyotyping and SNP array analysis. A run of homozygosity (ROH) was identified on chromosome 3 (3q11.2–q13.11). Although not diagnostic of a specific disorder, this finding suggested an increased risk for autosomal recessive conditions involving genes located within this region.

At four months, the patient underwent a first debulking surgical procedure with drainage and removal of colonic cysts. Complete resection was not feasible due to diffuse involvement of the entire intestine and mesenteric fan (Supplementary Figure 1). Histopathological examination confirmed the diagnosis of lymphangioma. Ten days postoperatively, the patient developed abdominal distension and irritability. Imaging studies excluded acute complications; however, antibiotics and intravenous immunoglobulins were administered in response to elevated inflammatory markers. Subsequent ultrasound monitoring revealed progressive enlargement of the known lymphatic cysts.

**Figure 4:**
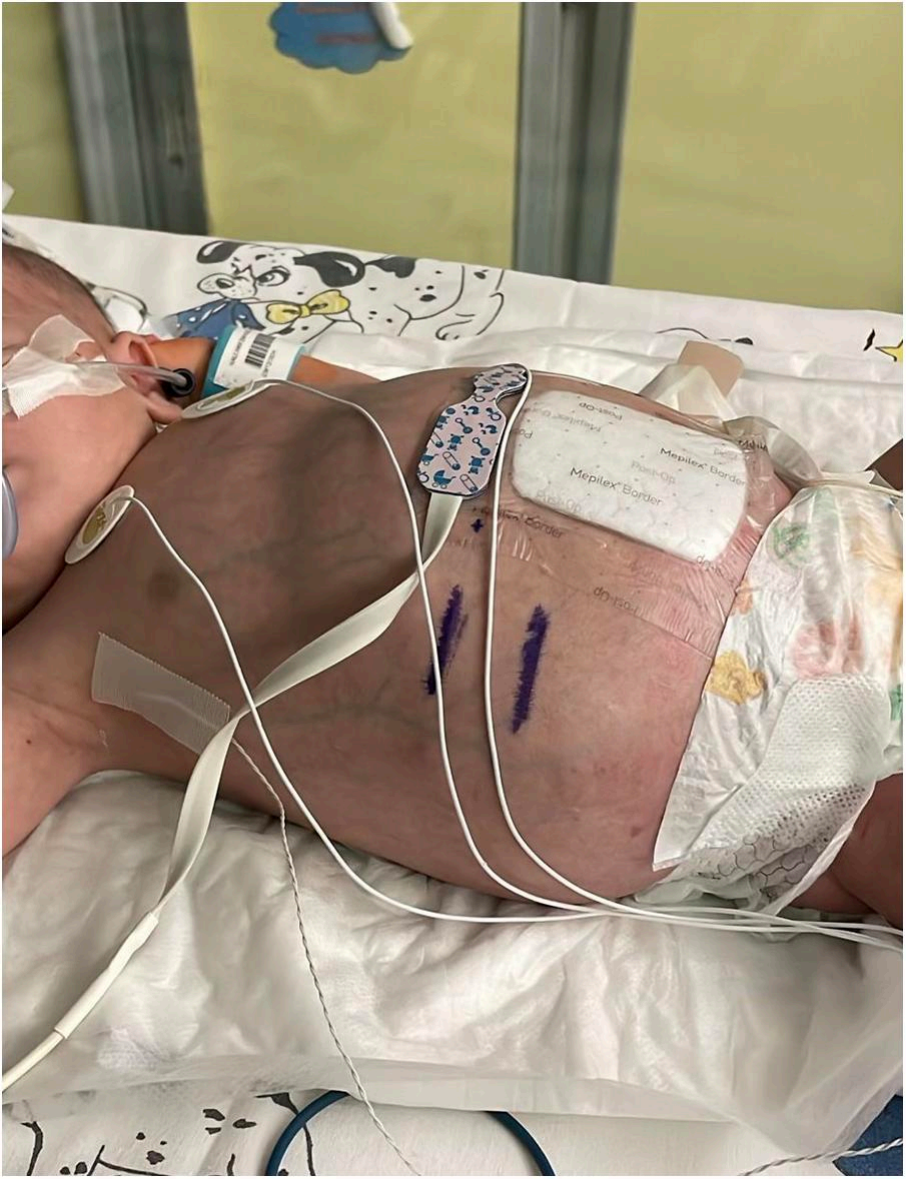
Infant abdomen appearance at few months old, showing marked distension due to the extensive subcutaneous lymphangioma (Bambino Gesù Children's Hospital, Rome, Italy).

**Figure 5:**
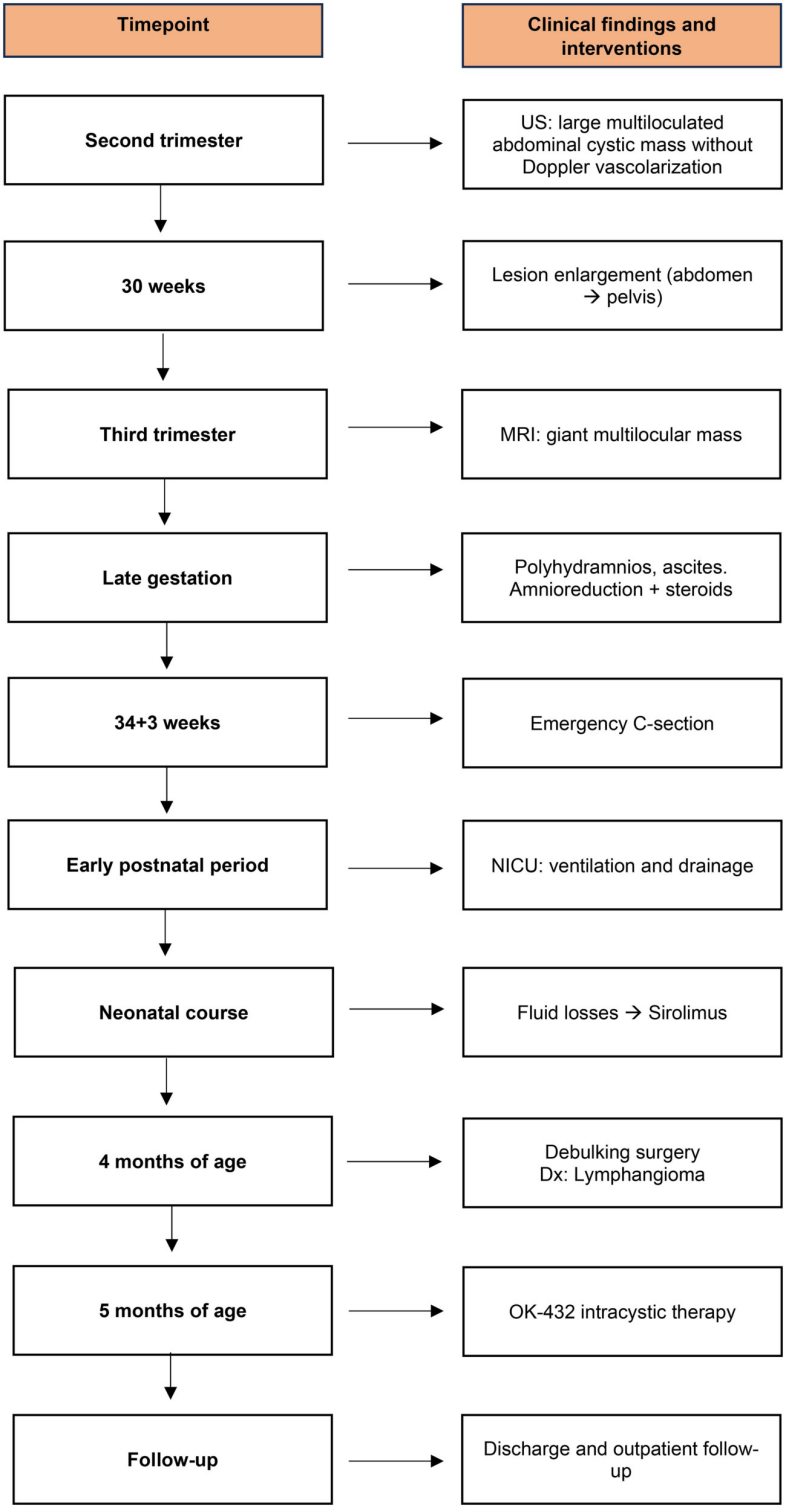
A timeline with relevant data from the episode of care.

At five months of age, persistent perisplenic and left paracolic gutter fluid collections were noted, reaching a maximum thickness of 41 mm. Following multidisciplinary discussion, intracystic therapy with OK-432 (Picibanil) at a dosage of 10 KE diluted in 10 mL was recommended and administered after aspiration of approximately 300 mL of cystic fluid. This intervention was followed by clinical deterioration and increased drainage of citrine fluid, necessitating transfer to the NICU for approximately one month (Figure 4). Ongoing ultrasound follow-up demonstrated persistent multiloculated abdominal lymphatic cysts and fluid collections, predominantly involving the iliac fossae and pelvis, without significant interval changes. Given the patient's clinical stability, the absence of further surgical indications and the need for prolonged nutritional support, he was transferred to the Nutritional Rehabilitation Unit. During this phase, abdominal circumference remained stable and satisfactory weight gain was achieved (from 7,735 g to 8,360 g), with no evidence of fluid reaccumulation. At 7 months of age, the patient was discharged in good general condition with instructions to continue nutritional and pharmacological therapy at home. A weekly outpatient follow-up program was established at the OPBG Gianicolo Unit, with close post-discharge monitoring. A timeline with relevant data from the episode of care is reported in Figure 5.

## 3 Material and methods

### 3.1 Case report

Written informed consent for publication of the case report and the clinical image was obtained from the patient's parents during the child's hospitalization at “Bambino Gesù Children's Hospital” in Rome, Italy.

### 3.2 Scoping review

A review protocol was developed *a priori* and the methodology was defined in accordance with the recommendations of the Preferred Reporting Items for Systematic Reviews and Meta-Analyses extension for Scoping Review (PRISMA-ScR) guidelines (8). The review was uploaded to PROSPERO (registration number: PROSPERO 2026 CRD420261286820) and may be accessed via this link: https://www.crd.york.ac.uk/PROSPEROFILES/2a563c93c2f6a1a0a5686a04fba7ced5.pdf. Given the rarity of fetal abdominal lymphangiomas and the predominance of case-based evidence, a scoping review methodology was therefore chosen to map existing knowledge, describe prenatal imaging features and identify patterns in clinical course, rather than to assess comparative effectiveness. Owing to the nature of this work, no approval from the Institutional Research Ethics Committee was required, as all data were extracted from previously published studies.

### 3.3 Search method

A systematic search for articles on the prenatal ultrasound diagnosis of abdominal and abdominopelvic lymphangiomas was conducted in PubMed, Scopus and Embase databases through September 2025. We included the studies from the earliest publication. No country restrictions were imposed. Only English-language fully published studies were considered.

### 3.4 Study selection

**Figure 6:**
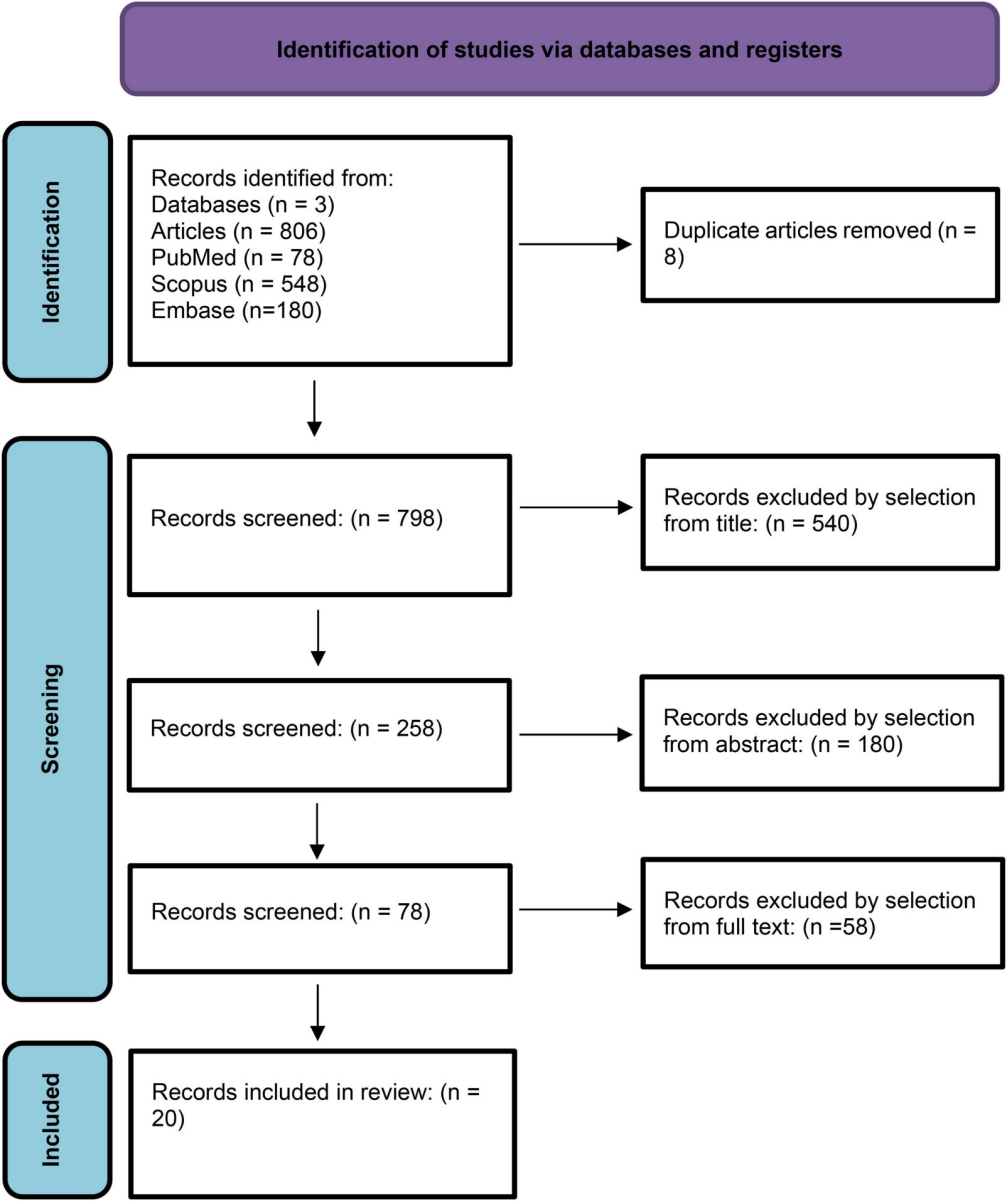
PRISMA-ScR flow chart.

Study selection was done independently by MG and MV. In case of discrepancy, IF decided for inclusion or exclusion. Inclusion criteria were: (1) studies that included prenatal ultrasound diagnosis and management of fetal abdominal lymphangiomas; (2) studies that reported at least one outcome of interest (neonatal outcome or postoperative course); (3) peer-reviewed articles, published originally. Non-abdominal localizations, postnatally diagnosed cases only, reviews and non-original studies, preclinical trials, animal trials and articles in languages other than English were excluded. *A comprehensive search was conducted using the following search string:* (“fetal” OR “prenatal”) AND (“abdominal” OR “mesenteric” OR “retroperitoneal”) AND “lymphangioma”. No geographic limits were applied. If possible, the authors of studies published only as congress abstracts were contacted via email and asked to provide their data. The studies selected and all reasons for exclusion are mentioned in the PRISMA flowchart (Figure 6). All included studies were assessed regarding potential conflicts of interest.

### 3.5 Quality assessment

**Figure 7:**
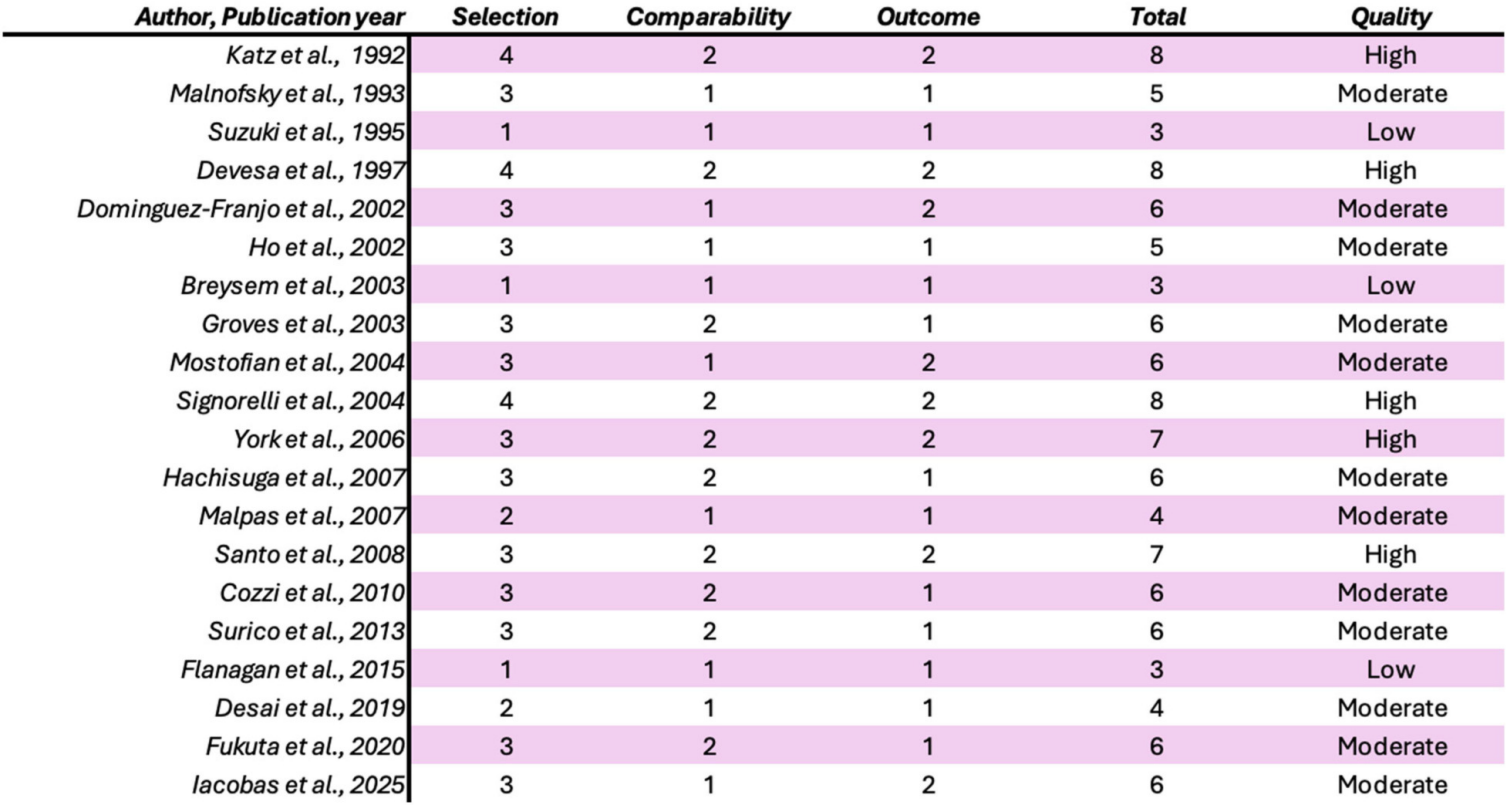
New Castle-Ottawa Scale (NOS).

Although critical appraisal is not mandatory for scoping reviews, study quality was assessed to contextualize the findings by using the Newcastle–Ottawa scale (NOS) (9). This scale comprises three broad factors (selection, comparability and exposure), with scores ranging from 0 (lowest quality) to 8 (highest quality). Two authors (MG and MV) independently rated the study's quality. Any disagreements were subsequently resolved through discussion or consultation with IF. NOS Scale is reported in Figure 7.

## 4 Results

### 4.1 Studies’ characteristics

After the database search, a total of 806 articles matched the search criteria. After removing records without full text and duplicates, 798 remained eligible. Of those, 540 records were excluded by selection from the title, 180 by selection from the abstract and 58 from the full text. Only 20 articles matched the inclusion criteria and were included in the scoping review. The countries in which the studies were conducted, the publication year range, the study designs and the numbers of participants are summarized in Supplementary Table 1. Overall, the publication years ranged from 1992 to 2025.

### 4.2 Outcomes

A total of twenty-one female patients with a mean age of 29.4 years, with a gestational age at diagnosis ranging from 18 to 39 weeks, were enrolled. Eleven cases were diagnosed in the third trimester, where the second-trimester anatomy scan revealed no abnormalities in all of them. In most cases, the mass was described as a multilocular, septated cystic, anechogenic in appearance and avascular on color Doppler imaging. The lesions ranged in size from 10 to 55 mm at diagnosis. In all cases, the mass increased in size during gestation. Regarding delivery outcomes, five pregnancies culminated in spontaneous vaginal delivery, one in operative vaginal delivery and twelve in cesarean section, most commonly due to cervical dystocia or breech presentation, with just one case performed for an acute abdominal condition in the fetus (10); in three cases, the information was not available. Cytogenetic analysis revealed no chromosomal abnormalities, except for a deletion on the long arm of chromosome 15, which was observed in a single case, excluded from this study because it did not meet the inclusion criteria (11). No additional structural abnormalities were reported at the time of the ultrasound scans, except for a case of jejunal atresia described by Fukuta et al. (12) a case of bilateral inguinal hernias reported by Katz et al. (13) and two cases of mild hydronephrosis (14, 15). Histopathological examination, when conducted, confirmed the diagnosis of a cystic lymphangioma. Surgical excision of the lesion was successfully performed in thirteen live-born neonates; postnatal sclerotherapy was administered in only two cases, whereas conservative treatment was adopted in the remaining two. In one additional case, in-utero therapy with maternal sirolimus administration was attempted. All the results are summarized in Supplementary Table 1.

When cases were analyzed according to lesion distribution and postnatal course, two clinically distinct patterns emerged. Focal abdominal lymphangiomas, typically confined to a single anatomical compartment and without diffuse mesenteric involvement, were more frequently amenable to complete surgical excision and associated with favorable neonatal outcomes. In contrast, diffuse lesions involving the mesentery or multiple intestinal segments were consistently related to persistent fluid accumulation, delayed or incomplete surgical management and prolonged hospitalization. Prenatal features such as progressive lesion growth, ascites and polyhydramnios were more commonly reported in these complex cases and were often associated with increased postnatal morbidity. Although formal predictive criteria are currently lacking, these findings suggest that prenatal imaging characteristics may help anticipate disease severity and support early multidisciplinary planning.

## 5 Discussion

Fetal abdominal lymphangiomas represent a significant diagnostic and therapeutic challenge due to their extreme rarity, anatomical complexity and unpredictable clinical course. Although lymphangiomas predominantly involve the cervicofacial region, abdominal localization is infrequently reported and is often detected incidentally during routine prenatal screening (15). The present case is notable not only for the lesion's unusual location and size, but also for its aggressive evolution, which necessitated prolonged, multimodal management.

Despite increasing recognition of fetal lymphangiomas through advances in prenatal ultrasound and fetal magnetic resonance imaging, abdominal and abdominopelvic localizations remain poorly characterized in terms of prognostic factors and standardized management strategies. In particular, limited guidance exists on how prenatal features - such as lesion size, diffuse mesenteric involvement, ascites, or polyhydramnios - should inform perinatal counseling, delivery planning, and postnatal therapeutic decision-making.

This case represents the most severe end of the clinical spectrum of fetal abdominal lymphangiomas**,** characterized by diffuse intestinal and mesenteric involvement, refractory fluid accumulation and limited responsiveness to conventional therapies. In contrast to the majority of reported cases, which are focal and surgically resectable, our patient exhibited features that substantially restricted curative options and necessitated a long-term, adaptive therapeutic strategy.

Comparison with cases included in the scoping review suggests that lesion distribution, rather than size alone, is a key determinant of outcome**.** Diffuse mesenteric involvement consistently limits the feasibility of complete resection and increases the risk of chronic complications, including chylous ascites, nutritional failure and recurrent inflammation. Accordingly, prenatal findings such as progressive lesion growth, ascites and polyhydramnios should raise suspicion for a more complex postnatal course**.**

Our patient was diagnosed prenatally with a large multiloculated abdominal mass complicated by polyhydramnios and ascites, both of which are recognized markers of poorer prognosis. Fetal MRI proved essential for anatomical characterization and perinatal planning. Despite the absence of associated structural anomalies or chromosomal abnormalities, the diffuse nature of the disease anticipated a challenging postnatal trajectory.

Recent reports, including the series by Iacobas et al. (2025), have demonstrated the feasibility of maternal sirolimus administration as an in-utero therapy for selected fetal vascular anomalies, with encouraging short-term results (16). In our case, prenatal treatment was precluded by rapid clinical deterioration and postnatal pharmacological and interventional approaches - including sirolimus, octreotide and sclerotherapy **-** provided only partial and transient benefit, underscoring the therapeutic limitations in extensive intestinal lymphangiomatosis.

The postnatal course was characterized by significant morbidity, including respiratory failure, persistent chylous ascites, infectious complications and prolonged nutritional dependence. Delayed debulking surgery contributed to partial stabilization and histological confirmation, but long-term improvement was achieved through sustained multidisciplinary management rather than a single definitive intervention**.**

Consistent with the findings of the scoping review, neonatal outcomes were more favorable when complete surgical resection was feasible**.** The absence of syndromic genetic abnormalities, including in our patient, supports the notion that fetal abdominal lymphangiomas are more often sporadic malformations. However, comprehensive genetic evaluation remains warranted in severe cases (17).

Overall, this case highlights the absence of standardized management strategies and the need for shared clinical frameworks. In the absence of evidence-based guidelines, individualized, multidisciplinary decision-making remains essential (18). Significantly, early recognition of diffuse disease may facilitate realistic counseling and a shift in therapeutic goals from curative intent toward long-term stabilization and quality-of-life preservation.

## 6 Conclusion

Fetal abdominal lymphangiomas constitute a rare but highly heterogeneous group of anomalies, in which the anatomical distribution and extent of involvement play a decisive role in determining the clinical course and therapeutic feasibility. This report highlights how diffuse intestinal and mesenteric disease profoundly limits curative surgical options and often necessitates a prolonged, multidisciplinary management strategy focused on stabilization rather than eradication.

Early prenatal diagnosis supported by advanced imaging remains essential for perinatal planning and multidisciplinary coordination, particularly in cases with extensive intra-abdominal involvement. Even in the presence of significant early morbidity and therapeutic setbacks, integrated and persistent care may allow long-term clinical stabilization and discharge.

The major challenge in this case was the diffuse involvement of the mesenteric fan, which precluded complete surgical excision and exemplifies a form of intestinal lymphangiomatosis associated with chronic inflammatory risk and long-term intestinal morbidity.

By integrating a complex clinical case with a scoping synthesis of the available literature, our findings emphasize the need for early recognition of high-risk features on prenatal imaging, transparent prognostic counseling and coordinated long-term care. The establishment of international registries and collaborative networks is essential to move beyond descriptive reporting toward evidence-informed frameworks that guide clinicians facing these challenging and uncommon conditions.

## Data Availability

The original contributions presented in the study are included in the article/Supplementary Material, further inquiries can be directed to the corresponding author.
