# Supplementary material for: Prenatal ultrasound diagnosis, intrauterine monitoring and postnatal management of a giant fetal abdominopelvic lymphangioma: a case report and scoping review
Source: Front Pediatr. 2026 May 7;14:1805856. doi: 10.3389/fped.2026.1805856 (PMC13189914; doi:10.3389/fped.2026.1805856)
Supplement: Supplementary file 1 [file Table1.docx]

| **Author** | Katz 1992 | Malnofsky 1993 | Suzuki 1995 | Devesa 1997 | Dominguez-Franjo 2002 | Ho 2002 | Breysem 2003 | Groves 2003 | Mostofian 2004 | Signorelli 2004 | York 2006 | Hachisuga 2007 | Malpas 2007 | Santo 2008 | Cozzi 2010 | Surico 2013 | Flanagan 2015 case a | Flanagan 2015 case b | Desai 2019 | Fukuta 2020 | Iacobas 2025 |
| --- | --- | --- | --- | --- | --- | --- | --- | --- | --- | --- | --- | --- | --- | --- | --- | --- | --- | --- | --- | --- | --- |
| **Journal** | American Journal of Perinatology | Journal of Ultrasound in Medicine | Journal of Maternal Fetal Investigation | Journal of Clinical Ultrasound | European Journal of Radiology | Journal of Ultrasound in Medicine | European Journal of Radiology | Ultrasound in Obstetrics and Gynecology | Journal of Ultrasound in Medicine | Prenatal diagnosis | Prenatal diagnosis | Fetal Diagnosis and Therapy | Prenatal diagnosis | The Journal of Maternal-Fetal and Neonatal Medicine | Fetal Diagnosis and Therapy | European Journal of Obstetrics and Gynecology | Pediatric Radiology | Pediatric Radiology | Journal of Fetal Medicine | International Journal of Surgery Case Reports | Prenatal Diagnosis |
| **Type of article** | Case report | Case report | Case report | Case report | Case report | Case report | Case series | Case report | Case report | Case report | Case report | Case report | Case report | Case report | Case report | Case report | Case series | Case series | Case report | Case report | Case series |
| **Country** | USA | USA | Japan | Spain | Spain | China | Belgium | USA | USA | Italy | USA | Japan | USA | Portugal | Italy | Italy | Germany | Germany | India | Japan | USA |
| **Maternal Age** | 26 years | 31 years | NA | 29 years | 35 years | 27 years | NA | 27 years | 31 years | 35 years | 30 years | 33 years | 23 years | 26 years | 34 years | 30 years | NA | NA | 24 years | 27 years | 32 years |
| **GA at diagnosis** | 28 weeks | 36.5 weeks | 27 weeks | 24 weeks | 30 weeks | 30 weeks | 39 weeks | 19 weeks | 25 weeks | 33 weeks | 24 weeks | 27 weeks | 18 weeks | 22 weeks | 26 weeks | 32 weeks | 31 weeks | 22 weeks | 28 weeks | 21 weeks | 25 weeks |
| **Size and charateristics of the cyst at the diagnosis** | Multicystic mass extending from the left lower abdomen to the left knee | Cystic septated mass in the left lower quadrant of the fetal abdomen, displacing the fetal bladder to the right | A well fined anechoic cyst was found in the fetal abdominal cavity | Infraumbilical and prevescical area unilocular anechogenic well delineated mass measuring 55x42x33 mm | Two abdominal cystic masses well defined, unicameral, echo-free, with no vascular signal at doppler colour flow mapping, measuring 42x41 and 52x21 mm and displacing kidneys posteriorly | A multilocular pelvic cyst, without blood flow, measuring 43x35x30 mm | Septated cystic lesion in right upper quadrant | Cystic mass in the abdomen | Multiple round and tubular anechoic spaces in the abdomen | Hyperechoic mass with heterogeneous tubular structures on the posterior aspect of the abdominal cavity | Cystic abdominal mass measuring 33x40x38 mm | Anechoic simple cyst, without blood flow, of 33x23x18 mm occupying the left retroperitoneum and extending from the edge of the left kidney almost to the bladder | Echogenic mass containing dilated loops of bowel | Right-sided abdominal mass of 23x18 mm anechogenic and septate with thin and irregular borders | A large intra-abdominal anechoic mass, resembling isolated fetal ascites, occupied nearly the entire fetal abdomen. It showed no definite septa but contained mild internal inhomogeneities and displaced the bowel loops posteriorly, compressing them | A multicystic mass of 30x34x25 mm, extending toward the left anterior abdominal wall, with well-defined borders and marked vascularization, but showing no continuity with intra-abdominal viscera and lacking solid components | Retroperitoneal hypoechoic cystic mass with internal septation. The diagnosis of lymphatic malformation, based on these images, was then confirmed postnatally | Retroperitoneal hypoechoic cystic lesion with internal septation. The diagnosis of lymphatic malformation, based on these images , was then confirmed postnatally | A huge multicystic, multiseptate and hypoechoic mass over sacral area with absence of flow on colour Doppler | A 10 mm cyst close to the lower pole of the right kidney | A large multicystic abnormality involving the lower left fetal trunk, gluteal region bilaterally, perineum, left retroperitoneum and left lower extremity |
| **Size and charateristics of the cyst at follow up** | No change in the ultrasonic appearance during all the sonographic studies | NA | At 35 weeks marked fetal intestinal dilation and ascites were noted | Following ultrasound examinations showed no changes until 32 weeks when fine septae were noted without an increase in size. By 39 weeks it increased in size to 68x50x37 mm and lateralized to the left | NA | The size of the pelvic cyst did not change on serial sonography | Retroperitoneal septated cyst extrarenal compatible with lymphangioma | Left sided septate lesion measuring 50x30x40 mm at 24 weeks, increasing to 90x10x40 mm at 37 weeks | At 30 weeks the mass was unchanged and it was suggestive of a lymphangioma or a hemangioma. By 35 weeks it decreased in size | No changes in the size and charateristics of the cyst | Follow-up ultrasound at 27 weeks revealed a large intra-abdominal multicystic abdominopelvic mass with solid components and vascularization in the cyst walls, extending into the right buttock and measuring 45×22×45 mm, suspicious for a sacrococcigeal teratoma. At 29 weeks the mass enlarged to 89x71x59 mm (intra-abdominal) and 58x41x33 mm (extra-abdominal) | Follow-up ultrasound at 29 weeks showed a progressive septation of the cyst, evolving to a honeycomb appearance by 33 weeks, suggestive of lymphangioma. The lesion gradually enlarged, reaching 47×39×27 mm at 38 weeks, without significant change in fluid echogenicity. MRI performed at 38 weeks showed a mass with features consistent with lymphangioma too | NA | At 37 weeks multilocular and multiseptate, measuring 78x58x77 mm and extending from the infrahepatic region to the vesical area | NA | In the follow up period, until delivery, no remarkable changes were noted in size, shape and texture of the cyst by serial ultrasound | NA | NA | At 34 weeks, the follow-up scan showed further progression of the lesion, which had enlarged to involve the upper thorax and back. Owing to its extensive distribution across multiple body regions, accurate measurement of the giant lymphangioma was not feasible | NA | NA |
| **Size and characteristics of the cyst at birth** | The mass extended from the left flank and ended sharply above the left knee. Magnetic resonance imaging confirmed the clinical diagnosis of lymphangioma | A postnatal sonogram confirmed the presence of a complex cystic mass measuring 73 x 34 x 37 mm, with multiple thin septations | NA | The neonatal ultrasound studies confirmed the prenatal findings that suggested an abdominal lymphangioma | NA | NA | NA | An MRI examination showed a very large (150x100x70 mm) apparently unilocular cystic mass with some septa, arising in the pelvis and extending into the upper abdomen | Bilobed, deformable, multiseptated, avascular cystic and solid mass in the right and left upper portions of the abdomen. The dominant mass on the right measured 37x25 mm and the mass on the left 41x17 mm | Multicystic mass containing serous fluid and arising from the greater omentum | MRI on day 1 revealed a heterogeneous, partially cystic mass anterior to the inferior vena cava (35x27 mm), extending inferiorly into the pelvis and right gluteal muscles, measuring 4 cm transversely and passing through the left sciatic notch into the left gluteal musculature | Ultrasound on day 1 confirmed the presence of a multiloculated left retroperitoneal mass of 47×39×27 mm. MRI on day 28, with Gd-DTPA showed enhancement of septal walls, confirming the diagnosis of a retroperitoneal lymphangioma | NA | NA | The lesion exhibited scanty thin septa and was primarily localized in the left abdominal quadrants, displacing the small intestine posteriorly. The bowel loops appeared non-dilated but surrounded by fluid | NA | NA | NA | Postnatal abdominal ultrasound revealed a large, multiloculated anechoic lesion with internal septa in the retroperitoneal region, extending to the lumbar, iliac, abdominal wall, and right lower thoracic wall, consistent with lymphangioma. MRI confirmed ultrasound findings | Proximal jejunal mesenteric cyst lymphangioma of 45 mm | A capillary‐venous‐ lymphatic malformation with extensive intra‐abdominal, pelvic and left lower extremity involvement, Klippel‐Trenaunay phenotype |
| **Histology of the cyst** | NA | Lymphangioma | NA | Cystic lymphangioma | Lymphangioma | Lymphangioma | NA | Lymphangioma | NA | Cystic lymphangioma | Lymphangioma | NA | Lymphangioma | NA | Lymphangioma | Lymphangioma | NA | NA | NA | NA | NA |
| **Abnormal karyotype** | Not performed for patients' will | None | NA | NA | NA | NA | NA | None | NA | 46, XX | NA | NA | None | 46, XX | NA | NA | NA | NA | NA | NA | NA |
| **Associated abnormalities** | Bilateral inguinal hernias | Mild right sided hydronephrosis | NA | None | None | None | NA | None | NA | Ascites | Mild bilateral hydronephrosis | None | Ascites and terminal ileum perforation | None | None | None | NA | NA | Subcutaneous edema involving lower part of thorax and the entire abdominal wall extending to right gluteal region. Mild to moderate hydronephrosis | Jejunal atresia | NA |
| **Pregnancy outcome** | C-section at 38w+5d for the suspect of a hemangioma | C-section | C-section for an acute abdominal condition in the fetus | C-section for fetal distress and cervical dystocia at 40 weeks | Vaginal delivery at term | Vacuum extraction at term | NA | Vaginal delivery at 39 weeks | Vaginal delivery at term | Vaginal delivery at 38 weeks and 2 days | C-section | C-section at 38 weeks for breech presentation | C-section at 38 weeks | C-section at 38 weeks | C-section at 38 weeks | C-section at 36 weeks for breech presentation and preterm premature rupture of membranes | NA | NA | C-section at 38 weeks | Vaginal Delivery at 32 weeks and 3 days | C-section at 37 weeks |
| **Neonatal outcome** | Surgical correction of the lymphangioma would have been evaluated over the first year of life if the lesion had not spontaneously regressed | A laparotomic excision of a left abdominal retroperitoneal mass extending deep into the pelvis was performed on day 10 of life | Laparatomic excision of a mass of 50 mm found in the mesentery of the jejunum involving a complicated volvulus | Laparotomic resction at 2 months | Surgical removal of the cystic masses at the first week of age. A mass attached to the jejunum was removed, with jejunal resection and end to end anastomosis. | Excision of the pelvic cyst was performed 5 days after birth | Conservative treatment | A laparotomic excision of the cyst arising from the sigmoid mesentery was performed, along with a sigmoidectomy, followed by a primary bowel anastomosis | Surgical removal at 2 months of a mesenteric lymphangioma compressing a short segment of the jejunum causing decreased bowel movements | Laparotomic resction at 10 days | The cystic mass was partially debulked to preserve bladder innervation. At 3 weeks, the buttock component enlarged and hardened, causing severe anemia; a second debulking was performed via a V-shaped sacral incision | At the time of the report, the infant was over 4 months old and he did not experienced any complications with the size of the mass unchanged | The large lympgangioma involving the terminal ileum and caecum was removed and an end-to-end anastomosis was performed | Laparotomic resction at day 6 | Laparotomic excision of the mass on day 14 of life accomplished along with segmental sigmoidectomy and primary anastomosis | Conservative managment until the baby was 3 months old because any cystic growth or change was evident in follow up clinical evaluations. After this time the baby underwent surgical excision of the lesion with no complications | Sclerotherapy interventions | Sclerotherapy interventions | Parents were reluctant for the treatment or surgical intervention | Surgical removal at day 2 | The infant experienced intermittent superficial bleeding from lymphatic vesicles, which served as entry sites for infection, resulting in two hospital admissions for cellulitis and intralesional bleeding at 12 and 14 months of age, despite ongoing systemic sirolimus therapy |
| **Post operative course** | NA | NA | NA | 18 months after the infant was asymptomatic without evidence of recurrence | Follow up showed no further abnormalities | A cystic lesion extending from the occipital area to the posterior neck was found 1 month later which was proven to be a lymphangioma by aspiration of lymphatic fluid from it | NA | Postoperative recovery was uneventful | Serial sonograms 2 years after surgery showed no residual or recurrent tumor | 4 months after the infant is completely asymptomatic | The patient had an uneventful recovery, was discharged on postoperative day 2 in good condition, and has since remained well without progression of the residual mass | NA | The infant developed recurrent chylous ascites and progressive lymphangioma despite multiple interventions, including a Ladd’s procedure and repeated peritoneovenous shunting. Persistent ascites caused respiratory compromise and the patient died at 23 months from overwhelming septicemia (H. influenzae and S. aureus) | NA | At 24 months’ follow-up, the infant is thriving well and remains recurrence-free | NA | NA | NA | Baby died on 20th postnatal day because of septic shock | NA | NA |

**Table 1.** Articles included in the scoping review
