# Supplementary material for: Prenatal ultrasound diagnosis, intrauterine monitoring and postnatal management of a giant fetal abdominopelvic lymphangioma: a case report and scoping review
Source: Front Pediatr. 2026 May 7;14:1805856. doi: 10.3389/fped.2026.1805856 (PMC13189914; doi:10.3389/fped.2026.1805856)
Supplement: Supplementary file 2 [file Table2.docx]

**Table 2.** A timeline with relevant data from the episode of care

**Clinical findings and interventions**

**Timepoint**

US: large multiloculated abdominal cystic mass without Doppler vascolarization

**Second trimester**

Lesion enlargement (abdomen 🡪 pelvis)

**30 weeks**

MRI: giant multilocular mass

**Third trimester**

Polyhydramnios, ascites. Amnioreduction + steroids

**Late gestation**

Emergency C-section

**34+3 weeks**

NICU: ventilation and drainage

**Early postnatal period**

Fluid losses 🡪 Sirolimus

**Neonatal course**

Debulking surgery

Dx: Lymphangioma

**4 months of age**

OK-432 intracystic therapy

Discharge and outpatient follow-up

**Follow-up**

**5 months of age**
