# Supplementary figures and images for: Prenatal ultrasound diagnosis, intrauterine monitoring and postnatal management of a giant fetal abdominopelvic lymphangioma: a case report and scoping review
Source: Front Pediatr. 2026 May 7;14:1805856. doi: 10.3389/fped.2026.1805856 (PMC13189914; doi:10.3389/fped.2026.1805856)

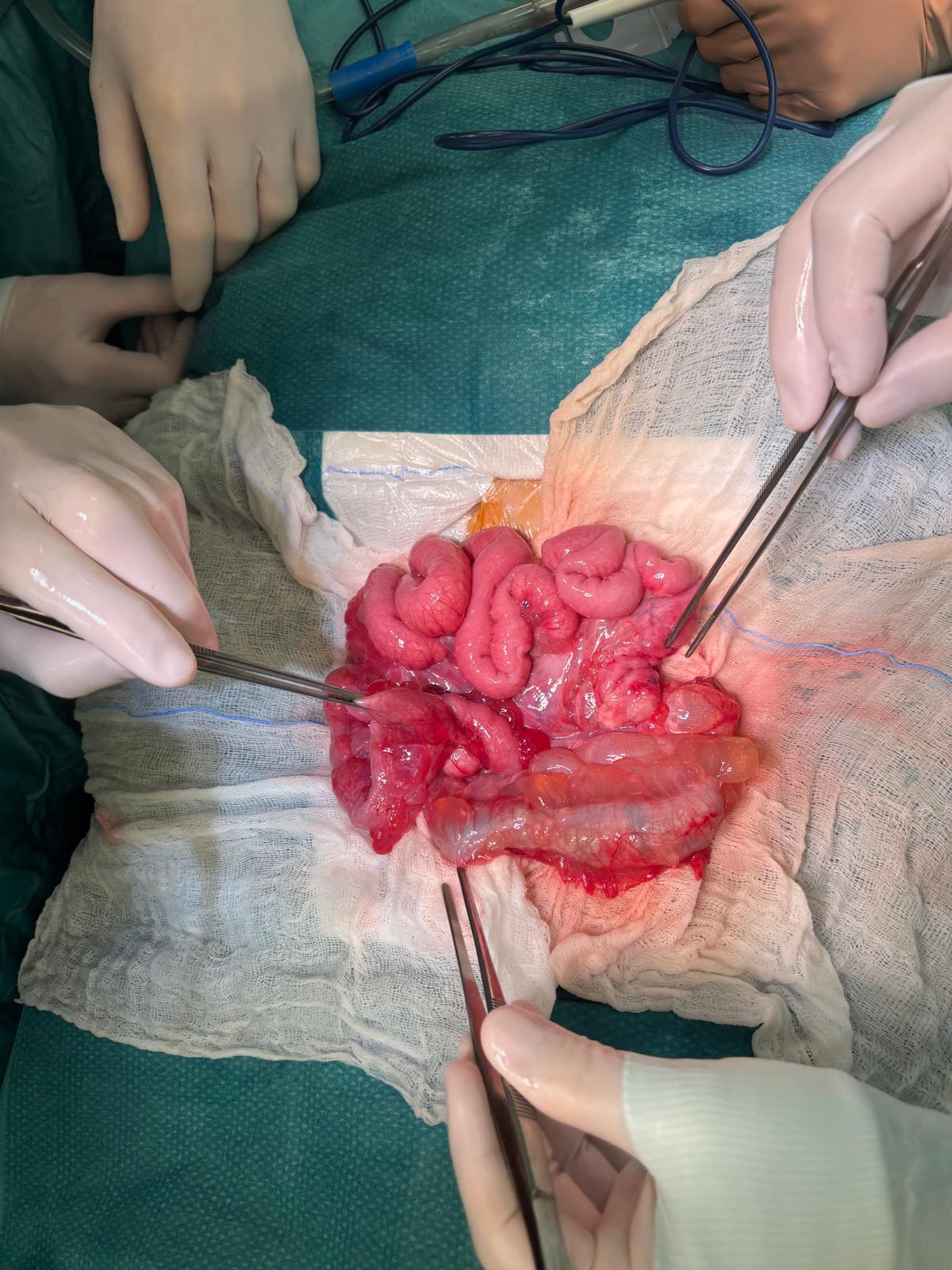

Supplement: Supplementary file 3 [file Image1.jpg]
